# Factors related to knowledge, attitudes, and behaviors regarding cervical cancer among Yemeni women

**DOI:** 10.1186/s12885-024-12435-y

**Published:** 2024-06-06

**Authors:** Boshra Ali, Andrzej Galbarczyk, Grazyna Jasienska, Maryam Ba-Break, Hülya Gül

**Affiliations:** 1 Public Health Department, Institute of Graduate Studies in Health Sciences, Istanbul University, Istanbul, Turkey; 2 Department of Environmental Health, Faculty of Health Sciences, Jagiellonian University Medical College, Krakow, Poland; 3 Nuffield Centre for International Health and Development, Leeds Institute of Health Sciences, Faculty of Medicine and Health, University of Leeds, Leeds, UK; 4 Department of Human Behavior, Ecology and Culture, Max Planck Institute for Evolutionary Anthropology, Leipzig, Germany; 5 Istanbul Faculty of Medicine, Public Health Department, Istanbul University, Istanbul, Türkiye

**Keywords:** Cervical cancer, Pap smear test, Education, Market integration, Yemen, Cancer prevention, Health literacy

## Abstract

**Background:**

Cervical cancer (CxCa), although preventable, is still among the most prevalent cancers in women. Mortality from this cancer is high, especially in low-income countries where preventive strategies are often lacking. We studied the knowledge, attitudes, and practices regarding CxCa among Yemeni women.

**Methods:**

This cross-sectional study was conducted in 2019 among 399 women in five major hospitals in Sanaa, the capital city of Yemen. Data were collected through face-to-face interviews using structured questionnaires. We used logistic regression models to analyze the likelihood of hearing about CxCa, believing that CxCa is treatable and preventable, awareness of the Pap smear test, and ever having this test, in relation to participant’s age, education level, working outside the household, and family history of CxCa.

**Results:**

Only 66.7% of the women had heard of CxCa. Women with higher education, working outside the household, and with a family history of CxCa were more likely to be aware of CxCa. Working outside the household was the only variable related to a higher likelihood of knowing that CxCa is a treatable and preventable. Furthermore, women with a family history of CxCa were more likely to have knowledge about Pap smear test and were more likely to have Pap smear test in the past.

**Conclusion:**

This study identified a low awareness of CxCa and its prevention among Yemeni women. In order to reduce the burden of CxCa in Yemen and save women’s lives, it is necessary to raise women’s awareness of this disease, especially among those with lower education and those not involved in work outside their homes.

## Background

Cervical cancer (CxCa) is the fourth most common and fatal type of cancer among women [1] and accounts for approximately 12% of all female cancers worldwide. It constitutes an important reproductive health problem and a major global health problem as woman die of CxCa every two minutes worldwide [2, 3]. According to estimates made by the World Health Organization (WHO), 20 million new cancer cases will develop globally by 2030, with 13 million deaths due to cancer [4–6]. Approximately 90% of the new cases and deaths in 2020 occurred in low- and middle-income countries (LMIC). This rate is 20 times higher than that in developed countries [7, 8]. Human papilloma virus (HPV) infection is one of the main causes of CxCa, while risk factors include smoking, socioeconomic status, steroid contraception, and sexual history [9, 10]. High-risk subtypes of the (HPV) cause nearly all cervical cancers, and HPV screening and vaccination programs are recommended effective strategies for disease prevention [11, 12].

Epidemiological studies have highlighted the importance of routine screening for CxCa prevention. Screening is influenced by many factors operating at the individual, family, and community level [3, 13]. Women’s knowledge and attitudes toward the disease are affected by sociodemographic factors. Most CxCa deaths occurred in women who had never been screened or treated. The programs carried out in developing countries are aimed at increasing the awareness of women, increasing the knowledge and skills of health personnel, and implementing effective monitoring and evaluation approaches [13]. The level of knowledge, attitude, and practice of society is very important in order to understand the signs of CxCa in a timely manner and to benefit from screening and early diagnosis [14]. The WHO and American Cancer Society recommend that eligible women should be screened for CxCa at least every three years [15, 16].

The scarcity of epidemiological data from Yemen makes it difficult to fully understand the health of this population. Currently, there are limited screening services and no national screening program in Yemen; therefore, most CxCa cases are detected at an advanced stage [17]. The importance of early detection is particularly important in a poor country like Yemen, where treatment services such as surgery, radiotherapy, and chemotherapy are often not available for advanced CxCa, early detection is particularly important. In the few institutions where these services are available and accessible, fees are often extremely high, and many women cannot afford them [18].

Education is one of the most important determinants of health and disease-preventive behaviors [3]. While the educational system in Yemen is very similar to that in most Arab countries, it differs in that education is not compulsory in Yemen, even though it was almost free in public schools before the war started in 2015. Yemeni society prioritizes the education of males, while females, if they are fortunate enough to be enrolled in school, rarely advance beyond the elementary level, which explains why the majority of women are illiterate. To eradicate illiteracy among women, the government has established literacy centers for women to teach them the principles of reading and writing. The high illiteracy rate among women hinders CxCa screening in low- and middle-income countries. However, this has been under-researched in Yemen.

### Objective

The aim of this study was to evaluate the knowledge, attitude, and prevention methods for CxCa among Yemeni women over the age of 15. This study also aimed to compare the knowledge and attitude regarding CxCa between women diagnosed with CxCa and those who were not diagnosed with any type of cancer. Moreover, we attempted to identify the factors that influence women’s knowledge and behavior. We hypothesized that the education level is the most important factor related to awareness level.

## Methods

### Study design

**Fig. 1:**
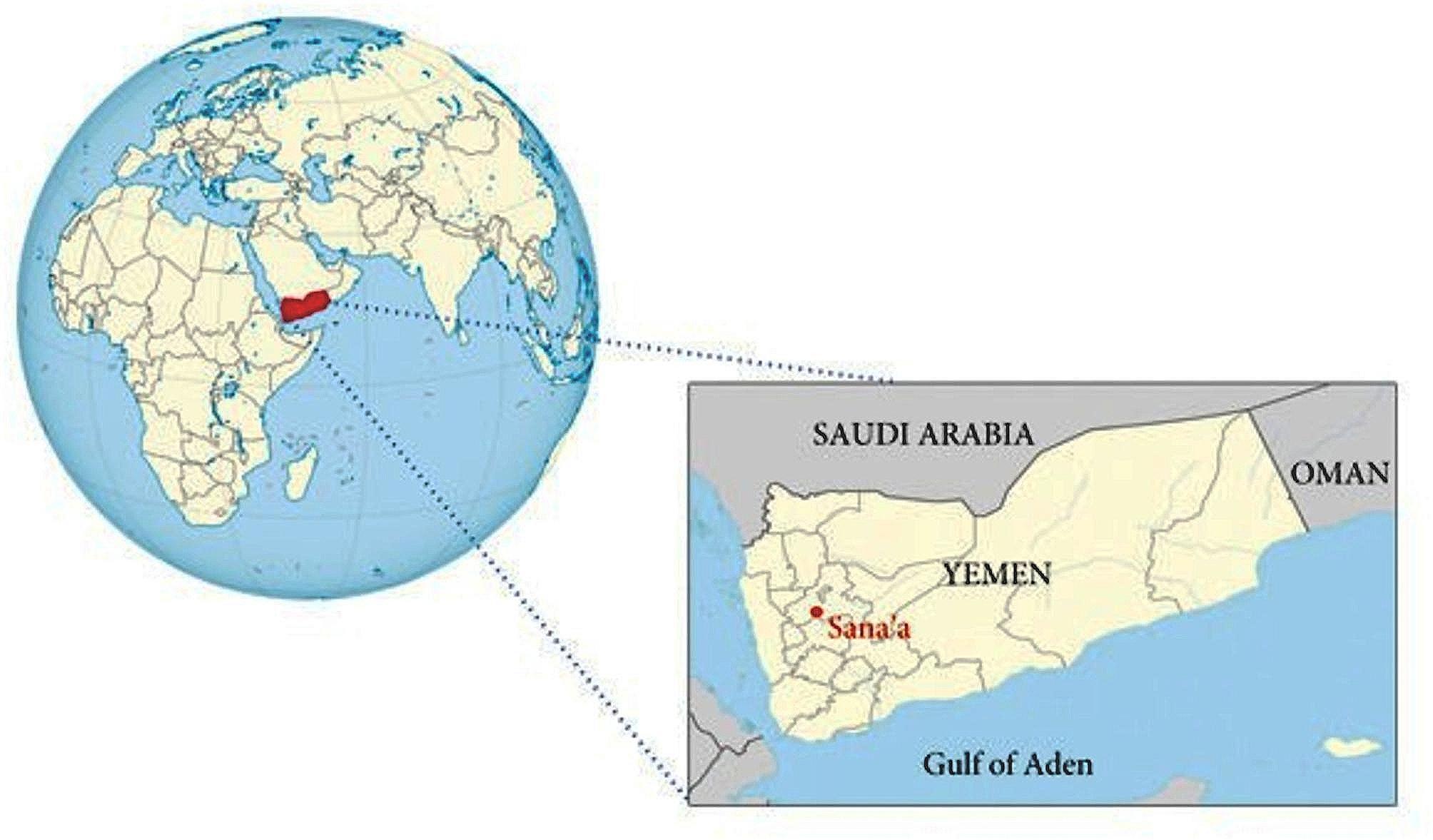
Location of the study area (Sana’a, Yemen)

This study had a cross-sectional design and was conducted between of April and December 2019 in Sana’a, the capital of the Republic of Yemen (Fig. 1). The study was conducted in accordance with the principles of the World Medical Declaration of Helsinki. After obtaining official research permission from the Ministry of Health in Yemen, we obtained permission from the ethics committee of Istanbul University, Faculty of Medicine. Ethical approval was obtained on April, 2019, and registered with number 81,007. Written informed consent was obtained from all participants.

### Sample size and sampling procedure

The sample group consisted of women living in Sana’a, with a population of 2,345,000 (1,299,000 men and 1,046,000 women) [19]. The study included women diagnosed with CxCa and those without CxCa. Cases diagnosed with CxCa were selected from the radiation and chemotherapy units of oncology centres. Patients who were admitted to gynaecological clinics (out-patient department) in the main public and private hospitals and were not diagnosed with any type of cancer were included in the non-cancer group. We recruited 100 women diagnosed with CxCa (including those undergoing chemotherapy or radiation therapy) and 299 non-cancer women. The study was conducted in three large public hospitals (i.e., Al-Thawra, Al Sabaeen and Al-Jumhori) and in Azal Private Hospital. Cases diagnosed with CxCa were selected from Yemen’s largest oncology center “National Oncology Center”, which covers more than five governorates according to the Ministry of Health, and Azal Hospital radiation center, using a random sampling method.

### Data sources and measurements

Data were collected using a structured questionnaire developed by the authors in English based on previous studies [20–22]. After piloting the questionnaire on 20 cases that were not included in the study, a final version of the questionnaire was prepared and unclear questions were corrected. Professional translators translated the questionnaire into Arabic. The questionnaire data were collected face-to-face by an Arabic native-speaking researcher. The women were then asked about age, education level, working outside the household, and family history of CxCa. Then women were asked if they had heard about CxCa and the Pap smear test. In the latter part, women were asked if they believed that CxCa is a preventable and treatable disease. Finally, we collected information about preventive behaviours by asking if women had taken a Pap smear test.

### Data management and analysis

Logistic regression (logit models) was used to determine the relationship between indictors of knowledge, attitude and behaviour about CxCa (i.e., hearing about CxCa, believing CxCa is treatable, believing CxCa is preventable, hearing about Pap smear test, and having Pap smear test) and age (*years*), education level (*Illiterate/Literate without formal education /Primary or secondary/University or equivalent*), working outside the household (*yes/no*), and family history of CxCa (*yes/no*). Each indicator was analysed using a separate logistic regression model. All analyses were conducted using Statistica version 13.3 (TIBCO Software Inc., Palo Alto, CA, USA).

## Results

The study group included 399 women from Sana’a, aged 15–70. The average age of the participants was 35.3 years (SD = 13.82). Women in the non- cancer group (mean 31.1, SD = 11.65) were significantly younger than those in the cancer group (mean 48.1, SD = 11.84, t=-12.58, *p* < 0.001). Regarding participants’ educational level, 32% of women were illiterate, and 7.5% could read but did not attend any school (Table 1). The majority of women (79.2%) worked outside the household and had no family history of CxCa (88.5%).

**Table 1:** Characteristics of the study participants. Differences between study groups (non- cancer cases and cancer cases) were tested by Χ^2^ tests

	All women *N* = 399		Non-cancer cases *N* = 299		Cancer cases *N* = 100			
	n	%	n	%	n	%	Χ^2^	p
Education								
Illiterate	126	31.6	69	23.1	57	57.0	42.07	< 0.001
Literate without formal education	30	7.5	22	7.4	8	8.0		
Primary or secondary	80	20.1	68	22.7	12	12.0		
University or equivalent	163	40.9	140	46.8	23	23.0		
Working outside the household								
Yes	316	79.2	228	76.3	88	88.0	6.27	0.012
No	83	20.8	71	23.7	12	12.0		
Family history of cervical cancer (CxCa)								
Yes	44	11.0	32	10.7	12	12.0	0.14	0.704
No	353	88.5	266	89.0	87	88.0		
Hearing about CxCa								
Yes	266	66.7	206	68.9	60	60.0	2.67	0.102
No	133	33.3	93	31.1	40	40.0		
Believing CxCa is treatable								
Yes	58	14.5	43	14.4	15	15.0	0.02	0.879
No	341	85.5	256	85.6	85	85.0		
Believing CxCa is preventable								
Yes	243	60.9	184	61.5	59	59.0	0.20	0.652
No	156	39.1	115	38.5	41	41.0		
Hearing about Pap smear test								
Yes	163	40.9	117	39.1	46	46.0	1.46	0.226
No	236	59.1	182	60.9	54	54.0		
Having Pap smear test								
Yes	71	17.8	53	17.7	18	18.0	< 0.01	0.951
No	328	82.2	246	82.3	82	82.0		

*Note*: CxCa refers to cervical cancer

Two-thirds (66.7%) of the participants had heard about CxCa, but only 14.5% believed that CxCa was treatable (14.5%). Although 60.9% believed that CxCa was preventable, more than half of the participants (59.1%) had never heard of the Pap smear test, and only 17.8% had a Pap smear test in the past (Table 1).

We performed a series of analyses to examine the factors affecting the level of knowledge, attitudes, and behaviors regarding CxCa. In the first step, we investigated the factors related to the likelihood of being aware of CxCa. Compared to illiterate women, only women with higher education were significantly more likely to be aware of CxCa (OR = 3.18, 95% CI = 1.62–6.25, *p* = 0.044). Furthermore, women who worked outside the household were over four times more likely to be aware of CxCa (OR = 4.68, 95% CI = 1.99–10.99, *p* < 0.001) than those who worked only in their homes. Women with a family history of CxCa were twice as likely to have heard about CxCa (OR = 2.11, 95% CI = 1.06–4.19, *p* = 0.033). Being diagnosed with CxCa and age were not related to the likelihood of being aware of CxCa (Table 2).

Next, we investigated the factors related to women’s attitudes towards CxCa and their belief that it is preventable and treatable. Only working outside the household was related to a higher likelihood of knowing that CxCa is a treatable, non-fatal disease (OR = 2.73, 95% CI = 1.36–5.51, *p* = 0.005), and preventable disease (OR = 3.48, 95% CI = 1.73–6.97, *p* < 0.001). The other factors did not have a statistically significant effect on the likelihood of believing that CxCa could be cured or prevented.

Finally, we examined the factors that influenced women’s awareness of the Pap smear test and whether they had ever undergone this test. Women with a family history of CxCa were more likely to have heard about the Pap smear test (OR = 2.86, 95%CI = 1.48–5.54, *p* = 0.002) and more likely to have this test (OR = 3.48, 95%CI = 1.73–6.97, *p* < 0.001) (Table 2).

**Table 2:** The likelihood of hearing about cervical cancer (CxCa), believing CxCa is treatable, believing CxCa is preventable, hearing about Pap smear test, and having Pap smear test in relation participant’s age, education level, working outside the household, and family history of cervical cancer. Results in bold are statistically significant (*p* < 0.05)

	Model 1 Hearing about CxCa				Model 2 Believing CxCa is treatable				Model 3 Believing CxCa is preventable				Model 4 Hearing about Pap smear test				Model 5 Having Pap smear test			
	OR	-95% CI	+ 95% CI	*p*	OR	-95% CI	+ 95% CI	*P*	OR	-95% CI	+ 95% CI	*p*	OR	-95% CI	+ 95% CI	*p*	OR	-95% CI	+ 95% CI	*p*
Age	1.00	0.98	1.03	0.694	0.97	0.94	1.00	0.077	1.01	0.99	1.03	0.568	1.00	0.98	1.02	0.914	1.01	0.99	1.04	0.374
Being diagnosed with CaCx	0.98	0.55	1.76	0.948	2.05	0.91	4.61	0.084	0.88	0.50	1.52	0.637	1.13	0.64	1.97	0.677	0.76	0.37	1.57	0.452
Education																				
Illiterate	*ref.*				*ref.*				*ref.*				*ref.*				*ref.*			
Lliterate without formal education	2.72	1.10	6.74	0.365	0.84	0.22	3.28	0.743	1.15	0.50	2.64	0.735	0.66	0.29	1.53	0.998	0.68	0.23	2.03	0.829
Primary or secondary	1.89	0.97	3.69	0.790	1.02	0.38	2.73	0.910	0.80	0.42	1.51	0.220	0.43	0.22	0.85	0.061	0.59	0.24	1.41	0.438
University or equivalent	**3.18**	**1.62**	**6.25**	**0.044**	1.09	0.44	2.72	0.715	1.26	0.67	2.36	0.337	0.68	0.36	1.26	0.928	0.76	0.34	1.68	0.938
Working outside the household	**4.68**	**1.99**	**10.99**	**< 0.001**	**2.73**	**1.36**	**5.51**	**0.005**	1.13	0.64	2.01	0.666	1.08	0.62	1.90	0.785	1.25	0.61	2.56	0.546
Having family history of CaCx	**2.11**	**1.06**	**4.19**	**0.033**	0.88	0.36	2.13	0.770	0.96	0.50	1.84	0.906	**2.86**	**1.48**	**5.54**	**0.002**	**3.48**	**1.73**	**6.97**	**< 0.001**

*Note* CxCa refers to cervical cancer, OR refers to odds ratio, CI refers to confidence interval

## Discussion

Although CxCa is preventable, treatable, and has a slow course with early diagnosis, the incidence of death due to this cancer is an important public health concern. Our results allowed us to identify several factors that influence the knowledge and attitudes of Yemeni women about CxCa. We showed that being diagnosed with CxCa did not affect knowledge regarding CxCa, which may seem surprising and counterintuitive. However, it should be noted that it is common in Yemen to not inform patients about having cancer. When we conducted our study, we were frequently requested by a relative of the participant to not mention to her that she had already been diagnosed with CxCa. We were able to do interviews with some patients only under this condition. When we asked relatives for the reason to conceal such a fact, they said, “If patients know about diagnosis, they would refuse the treatment and their psychological well-being would be negatively affected”. Similar situation was identified in other LMICs [23].

Similarly, it might be surprising how little Yemeni women’s knowledge about CxCa depends on their educational level. Our findings showed that only women with a university education were more aware of CxCa than illiterate women. This might be explained by the poor health-related content of the Yemeni school curriculum, even before the war, since the education system had never been adequate. After the war, the country lost the opportunity to improve its school system [24]. Furthermore, women’s education has never been prioritized in this country. Moreover, Yemeni culture considers discussing women’s reproductive health with young and unmarried women taboo. Therefore, topics related to reproductive health are not included in the school curricula. Therefore, the lack of health-related topics in school curricula might explain our findings that educational level does not predict the knowledge or attitudes of women about CxCa. Similar findings have been reported for many LMICs [25, 26].

According to our findings, women with a family history of CxCa are more likely to be aware of and use preventive measures against this disease. Even if the woman herself is not aware of having cancer, her relatives like daughters, sisters etc. have a chance to hear about it and begin to seek more information about the disease. A family history of CxCa is obviously not a modifiable factor that would be useful from a health-promotion perspective. However, more publicity can be given to the stories of women who have suffered from this disease, especially stories describing the positive aspects associated with early diagnosis and successful treatment. In order to encourage women to utilize screening services, these stories could be shown in mass media and educational channels [27]. These stories could also be shown on televisions in waiting rooms, which are common in Yemeni hospitals.

We have also shown that working outside the household was a factor related to having better knowledge of CxCa. These results might be interpreted within the context of market integration. Market integration is a process that involves transition from agricultural activities to occupations that are not directly related to subsistence, such as market work or education-based employment, but also involves broader economic and sociocultural changes that accompany this process [28]. The market integration process leads to health and environmental changes. It has been previously shown that market integration might influence population health and well-being, and, as our study has shown, the level of health awareness [29, 30].

To the best of our knowledge, our study provides the first data on the factors related to the knowledge, attitudes, and practices regarding CxCa among Yemeni women. In similar studies, the knowledge of women regarding CxCa was higher than in our study. In Nigeria, all (100%) participants had heard of CxCa [31], and similarly, in Gabon, 91.6% of women stated that they heard of this disease [6]. In Laos, in a study with female health workers, a rate of 89.9% was observed [32]. Compared to these results, Yemeni women’s knowledge seems to be very low, with only 66% having previously heard of CxCa.

In our study, 85% of women believed that CxCa was not treatable. In study conducted in Ethiopia only a quarter of 583 women had the same beliefs [20]. In our study, 60.9% of the women were aware that the prevention of CxCa was possible. In a study conducted in Malaysia this rate was 74.4% [33]; in Nigeria, it was 95.2% [31], while in Ethiopia, only 50% of the participants were aware that cancer is preventable [20].

Only 39.1% of Yemeni women in the non-cancer group and 46% of the cancer group had heard about the Pap smear test. The closest result to our study was observed in Cambodia (34%) [5]. A higher rate is observed in high-income countries. By comparison, Italy had the highest level of knowledge, with 92% of women [34], while more than half (55.2%) of students in South Africa [22] and only 2% of women in India heard about this test [4]. Relating the level of Pap smear testing, it was found that 95% of Swedish women [35], 93% of US women [36], and 67% of Nigerian women [27] had been examined at least once in their lifetime. Our study revealed that only 17.7% of women in non-cancer group and 18% of women in cancer group were ever tested. Even lower testing was found in South Africa, where only 3.2% of 1546 women had a Pap smear test [37].

## Limitations

Because of the war in Yemen, the current security situation, and political problems, conducting a study is challenging. In addition, due to cultural taboos associated with sexual health, some women refused to be interviewed or chose not to answer some questions. Errors that may arise from communication, such as exaggerated or incorrect answers by participants, are also considered as limitations. Therefore, our study group was not representative of all the Yemeni women. Women who participated were recruited from hospitals and health clinics. Consequently, we expect an even lower level of knowledge in the general population. On the other hand, we were able to interview a large number of women (*N* = 399) of diverse ages, education levels, and family situations and included women with CxCa and women who were free of this disease.

## Conclusion

A low level of awareness of CxCa and prevention methods was observed among Yemeni women. These results could be explained by the inaccessibility of health services in economically underdeveloped countries as the low level of knowledge was similar to that in other developing countries. It is well documented that women’s knowledge regarding CxCa, especially about the fact that it is preventable and treatable, increases the chance that women will become interested in prevention. Thus, identifying this lack of awareness and knowledge is a crucial first step in implementing a successful CxCa prevention program that focuses on increased screening practices.

We showed that education in Yemen does not significantly improve Yemeni women’s awareness of CxCa, except for university education, and even this only for selected aspects of knowledge. Importantly, our results indicate that market integration in Yemen might be more strongly related to having a knowledge about CxCa than education. This suggests that while sexual health education in school is necessary, introducing such programs could be difficult or even impossible given the cultural taboos that prevent discussion of sex-related topics among women who are not married. Public health programs should, therefore, focus not only on targeting women in workplaces, but also on reaching women who are not working outside their homes, since these women have the lowest level of knowledge and thus a high risk of not taking advantage of prevention strategies.

## Data Availability

The datasets used and/or analyzed during the current study are available from the corresponding author on reasonable request.
